# HMOX1 mRNA combined with early imaging parameters for predicting the prognosis of hypertensive cerebral hemorrhage patients undergoing conservative treatment

**DOI:** 10.3389/fneur.2025.1657188

**Published:** 2025-12-01

**Authors:** Jinjiang Dong, Xiaofeng Ye, Popo Wang, Qijun Ye, Deqing Peng

**Affiliations:** 1 Department of Neurosurgery, Chun'an First People's Hospital, Hangzhou Medical College Affiliated Chun'an Hospital, Hangzhou, China; 2 Center for Rehabilitation Medicine, Department of Neurosurgery, Zhejiang Provincial People's Hospital (Affiliated People's Hospital, Hangzhou Medical College), Hangzhou, Zhejiang, China

**Keywords:** hypertensive cerebral hemorrhage, HMOX1, imaging parameters, prognosis, nomogram model

## Abstract

**Objective:**

To address the limitations of current clinical tools in predicting the prognosis of hypertensive cerebral hemorrhage (HICH) patients undergoing conservative treatment, this study aimed to evaluate the predictive value of combining HMOX1 mRNA levels with early imaging parameters.

**Methods:**

This retrospective diagnostic accuracy study involved 208 HICH patients (mean age 50 ± 9.5 years, 149 men) treated from December 2022 to December 2024. Patients were divided into good prognosis (mRS score ≤ 2, *n* = 112) and poor prognosis (mRS score > 2, *n* = 96) groups based on 90-day mRS scores. The index tests were HMOX1 mRNA levels and early imaging parameters (hemorrhage location, hematoma volume, and shape), with the reference standard being the 90-day mRS score. Statistical analyses included hierarchical regression, multivariate logistic regression, and ROC curve evaluations. Bootstrap resampling (1,000 samples) confirmed that the OR values of key risk factors were stable.

**Results:**

HMOX1 mRNA expression was higher in the poor prognosis group (1.36 ± 0.41 vs. 1.00 ± 0.22, *p* < 0.001). Delong test showed that the AUC of the combination of HMOX1 mRNA and imaging parameters (0.871, 95% CI: 0.818–0.924) was significantly higher than that of single indicators (all *p* < 0.05). A nomogram model incorporating these factors plus clinical and laboratory indicators showed good discrimination (*C*-index: 0.814) and calibration (AUC: 0.94), and its AUC was significantly higher than that of the above combined indicator (*p* = 0.003).

**Conclusion:**

The ‘HMOX1 mRNA + imaging parameters' combined indicator and the nomogram model both significantly improve prognostic prediction accuracy in HICH patients undergoing conservative treatment, with the nomogram model showing the best efficiency.

## Introduction

Hypertensive cerebral hemorrhage (HICH) is a critical neurological condition with high mortality and disability rates, accounting for 30%−60% of all strokes (1). Current clinical prognosis assessment tools, such as the Glasgow Coma Scale (GCS), the National Institutes of Health Stroke Scale (NIHSS), and the modified Rankin Scale (mRS), are limited by subjectivity and fail to comprehensively reflect the pathological state, particularly the role of oxidative stress and inflammatory responses in secondary brain injury (2–4). Recent research has highlighted the role of ferroptosis, a form of iron-dependent programmed cell death, in secondary brain injury following cerebral hemorrhage (5, 6). Heme oxygenase 1 (HMOX1), a key enzyme in heme metabolism, has been shown to play a pivotal role in regulating ferroptosis after cerebral hemorrhage (7, 8). Animal experiments indicate that upregulating HMOX1 expression can inhibit ferroptosis, reducing neuronal injury (7). However, the relationship between HMOX1 mRNA expression and patient prognosis in HICH remains unclear.

The purpose of this investigation is to evaluate the predictive value of combining HMOX1 mRNA levels with early imaging parameters for the prognosis of HICH patients. Previous studies have explored the use of imaging parameters such as hematoma volume and shape, but these features alone cannot fully capture the patient's pathological and physiological state (8, 9). Additionally, while the role of ferroptosis in secondary brain injury has gained attention, few studies have systematically investigated the combined predictive value of HMOX1 mRNA and imaging parameters. This study addresses this gap by exploring the combined predictive potential of HMOX1 mRNA and early imaging parameters, aiming to provide a more accurate and objective prognosis assessment tool for HICH patients.

To solve this problem, we plan to use a combination of HMOX1 mRNA levels and early imaging parameters to develop a comprehensive prediction model. We will measure HMOX1 mRNA levels in peripheral blood and analyze early imaging parameters, including hemorrhage location, hematoma volume, and shape, using CT scans. By integrating these molecular and imaging markers with clinical data, we aim to construct a nomogram model that can provide a more precise and individualized prognosis prediction for HICH patients. This approach is expected to improve the accuracy of early risk stratification for poor prognosis, offering a scientific basis for developing targeted neuroprotective therapies and individualized treatment plans.

## Materials and methods

### Study design and location

This retrospective study was approved by the Institutional Review Board (IRB) of Chun'an First People's Hospital, and conducted in accordance with the ethical principles of the Declaration of Helsinki. Written informed consent was obtained from all patients or their legal representatives. Patient data were retrieved from our hospital's neurosurgery department for the period from December 2022 to December 2024. The study involved a consecutive selection of patients who met the inclusion criteria.

### Participants

A total of 208 HICH patients were included in this study. It is important to note that none of the patients included in this study experienced hematoma rupture into the ventricle. Inclusion criteria were: (1) aged 18–80 years; (2) met the diagnostic criteria for HICH and underwent conservative treatment; (3) completed cranial CT scanning within 24 h of onset and cranial CT review on day 7; (4) were initially diagnosed with HICH or secondary ventricular hemorrhage due to HICH; (5) had complete clinical data; and (6) were followed up for 3 months via outpatient visits or telephone. Exclusion criteria were: (1) patients with ventricular hemorrhage combined with subarachnoid hemorrhage; (2) patients with secondary cerebral hemorrhage due to trauma, cerebral venous thrombolysis hemorrhagic transformation, cerebral tumors, intracranial aneurysms, or arteriovenous malformations; (3) patients with poor-quality cranial CT images; (4) patients with systemic diseases such as cancer, heart failure, or severe renal insufficiency; and (5) patients who abandoned treatment or died rapidly during hospitalization due to severe illness.

**Figure 1:**
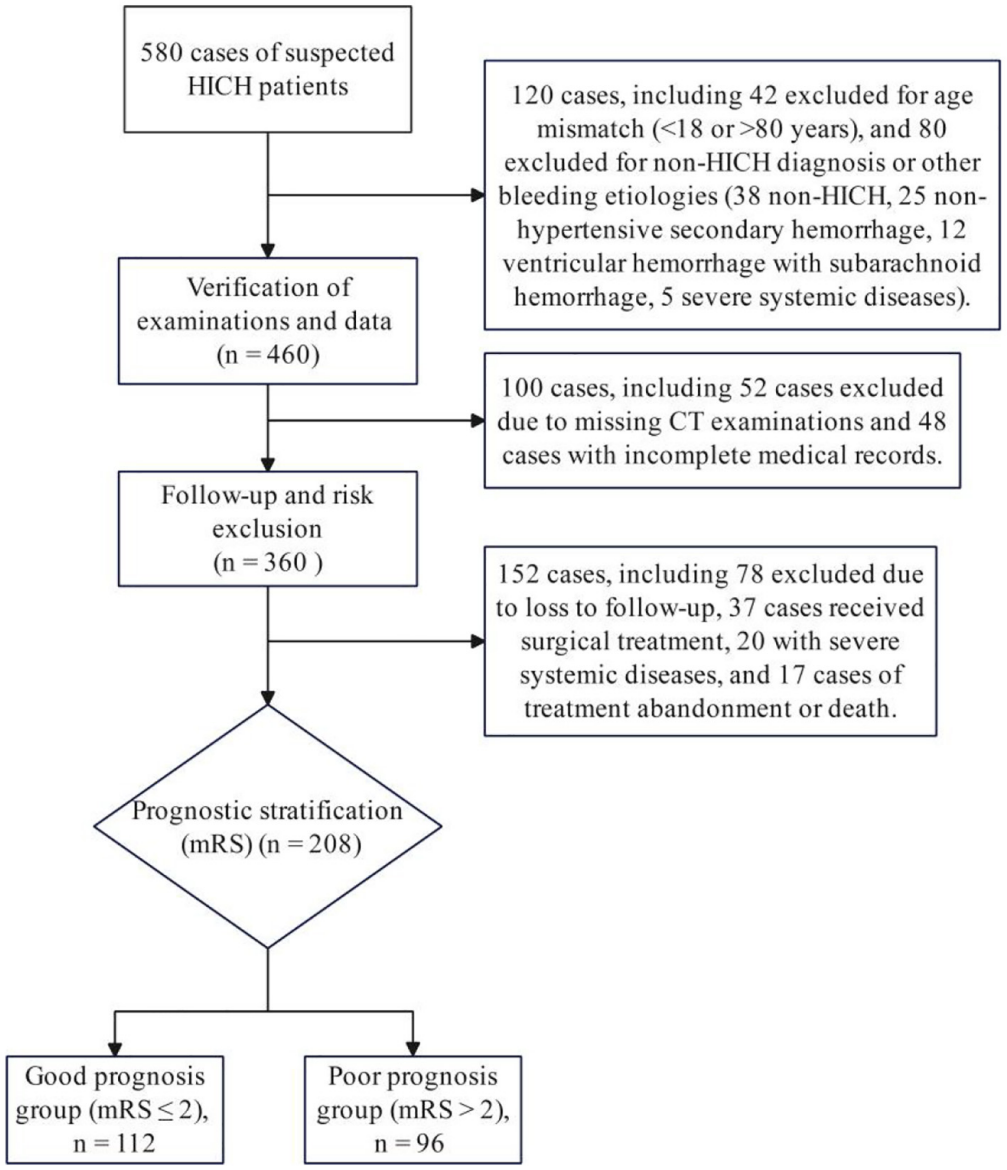
Flowchart for screening and prognostic assessment of HICH patients.

To clearly illustrate the screening process for the study participants, a flowchart of participant screening (Figure 1) has been developed. This flowchart details the screening pathway for all hypertensive intracerebral hemorrhage (HICH) cases (total of 580 cases) admitted to the Department of Neurosurgery of our hospital from December 2022 to December 2024. Among these cases: 42 cases were excluded for failing to meet the age criteria (< 18 or >80 years); 80 cases were excluded for non-HICH diagnosis or other bleeding etiologies (including 38 non-HICH cases, 25 non-hypertensive secondary hemorrhage cases, 12 ventricular hemorrhage with subarachnoid hemorrhage cases, and 5 severe systemic disease cases); 52 cases were excluded due to missing CT examinations (not completing cranial CT within 24 h of onset or follow-up CT on day 7); 48 cases were excluded with incomplete medical records; 37 cases were excluded for receiving surgical treatment; 17 cases were excluded for treatment abandonment or rapid death during hospitalization. Finally, 208 HICH patients receiving conservative treatment who met all criteria were included in the study.

### Index test and reference standard

The index tests in this study were the quantitative measurement of HMOX1 mRNA levels in peripheral blood and the assessment of early imaging parameters, including hemorrhage location, hematoma volume, and shape. HMOX1 mRNA levels were measured using reverse transcription quantitative polymerase chain reaction (RT-qPCR). Blood samples were collected within 24 h of admission. Total RNA was extracted using a commercial kit (TIANamp Blood RNA Kit, TianGen). cDNA was synthesized using a reverse transcription kit (PrimeScript RT Master Mix, Takara), and RT-qPCR was performed using a SYBR Green-based qPCR kit (TB Green Premix Ex Taq, Takara) on a qPCR instrument (QuantStudio 7, Thermo Fisher Scientific). The imaging parameters were assessed using baseline cranial CT scans retrieved from our hospital's imaging department. The CT scans were performed using a GE Healthcare CT scanner, with imaging parameters set at 120 kV and 250 mA. The reference standard was the 90-day mRS score, which was assessed during outpatient follow-up. The mRS score was determined by a neurologist through clinical evaluation.

### Evaluation procedures

DICOM data from the baseline cranial CT scans were imported into the 3D Slicer software (version 5.7.0). Cranial CT images were assessed using brain tissue windows, set at a window level of 35 Hounsfield Units (HU) and a window width of 80 HU. The red slice-only module was chosen, and the Reformat plugin was utilized to adjust the head position. Marking tools were used to measure the distance of each diameter, and the accuracy of hematoma segmentation was confirmed through visual inspection. In cases where the intraparenchymal hematoma was connected to the intraventricular blood, manual segmentation was performed. The segmentation component was then used to send the segmented hematoma data for 3D imaging and viewing. The hematoma volume, along with the hemorrhage location and hematoma shape, was recorded. Two neurosurgeons, blinded to the data, measured the results, and the average value was taken. In cases of significant disagreement, group discussions were held to reduce measurement bias.

### Observation indicators

For each patient, the following observation indicators were recorded: general information, including gender, age, body mass index (BMI), smoking status, drinking history, diabetes history, hyperlipidemia history, and hospitalization days; admission symptoms and vital sign assessment, including total National Institutes of Health Stroke Scale (NIHSS) scores and Glasgow Coma Scale (GCS) scores; laboratory values, including white blood cell count, absolute neutrophil count (ANC), absolute lymphocyte count (ALC), absolute monocyte count (AMC), hemoglobin, platelet count, fasting blood glucose, creatinine, urea nitrogen, hypersensitive C-reactive protein (hs-CRP), D-dimer, and fibrinogen degradation products (FDP), all obtained from routine blood and biochemical tests within 24 h of admission; baseline imaging parameters, including hemorrhage location, hematoma volume, and hematoma shape, assessed from cranial computed tomography (CT) scans; and peripheral blood HMOX1 mRNA expression levels at admission.

### Follow-up, prognosis assessment, and grouping

Patients were followed up at outpatient clinics 90 days after onset, and their prognosis was assessed via the mRS scale, with scores ranging from 0 to 6 (0 indicating no symptoms and 6 indicating death). A score of ≤ 2 was defined as a good prognosis, and a score of > 2 was defined as a poor prognosis (10). Patients were divided into a good prognosis group (*n* = 112) and a poor prognosis group (*n* = 96).

### Statistical methods

Data analysis was performed via SPSS 23.0 software (IBM, USA). Continuous data that followed a normal distribution were expressed as mean ± standard deviation (*x* ± *s*), and intergroup comparisons were performed using independent-samples *t*-tests. Categorical data were presented as rates (%), and intergroup comparisons were conducted using the chi-square (χ^2^) test or Fisher's exact test (when cell counts were < 5).

To explore the associations between clinical characteristics and modified Rankin Scale (mRS) scores, we employed hierarchical regression analysis. Multivariate logistic regression analysis (using the Enter method) was used to identify independent factors influencing poor prognosis in patients with HICH. A nomogram model was constructed using R 3.4.3 software, and its calibration performance was evaluated via a calibration curve.

Receiver operating characteristic (ROC) curves were utilized to assess the prognostic predictive efficacy of HMOX1 mRNA alone, early imaging parameters alone, their combination, and the nomogram model for poor prognosis. To determine whether differences in the area under the ROC curve (AUC) between different predictive indicators/models were statistically significant, pairwise comparisons were performed using the Delong test (implemented via the “pROC” package in R 3.4.3). This test was used to verify whether: (1) the combined indicator of “HMOX1 mRNA + early imaging parameters” outperformed individual indicators; and (2) the nomogram model outperformed both the combined indicator and individual indicators.

The Hosmer–Lemeshow (H–L) test was used to quantitatively evaluate the nomogram's calibration. Patients were stratified into *10* groups based on their predicted probability of poor prognosis, and the discrepancy between the predicted and actual incidence of poor prognosis was calculated. A non-significant H–L chi-square (χ^2^) statistic (*P* > 0.05) indicated good calibration of the model.

Decision curve analysis (DCA) was conducted to assess the clinical utility of the model. The x-axis represented the threshold probability (the risk threshold at which clinicians decide to intervene), and the y-axis represented the net benefit (NB)—defined as the benefit of correctly identifying high-risk patients minus the harm of incorrectly classifying low-risk patients as high-risk. The net benefit of the nomogram-based multivariable model was compared with that of the HMOX1 mRNA-only model, hematoma volume-only model, and two extreme clinical strategies (“Treat All” and “Treat None”). The criterion for superior clinical utility was a higher net benefit within the clinically relevant threshold probability range (0.01–0.5).

The H–L test was performed using SPSS 23.0, and DCA was implemented via the “rmda” package in R 3.4.3. A two-tailed *P*-value < 0.05 was considered statistically significant for all analyses.

## Results

### Comparison of clinical data between the good and poor prognosis groups

Compared with the good prognosis group, the poor prognosis group had significantly greater NIHSS scores, hs-CRP levels, proportions of hemorrhages in the cerebral lobe and thalamus, hematoma volumes, and proportions of irregular hematoma shapes. GCS scores and creatinine levels were significantly lower in the poor prognosis group (*P* < 0.05) (Table 1). The observed GCS scores indicate mild to moderate impairment in consciousness, while the higher NIHSS scores reflect more severe cumulative neurological deficits across multiple domains assessed by the scale, such as motor function, language, and sensory perception, which are common in intracerebral hemorrhage.

**Table 1:** Comparison of clinical data between the good and poor prognosis groups.

**Group**	**Good prognosis group (*n* = 112)**	**Poor prognosis group (*n* = 96)**	***t*/χ^2^**	***P***
**Gender**				
Male	86 (76.79)	63 (65.63)	3.169	0.075
Female	26 (23.21)	33 (34.38)		
Age (years)	49.62 ± 10.07	50.28 ± 8.92	0.497	0.620
BMI (kg/m^2^)	24.97 ± 2.31	25.05 ± 2.26	0.251	0.802
**Smoking history**				
No	72 (64.29)	57 (59.38)	0.529	0.467
Yes	40 (35.71)	39 (40.63)		
**Drinking history**				
No	57 (50.89)	55 (57.29)	0.852	0.356
Yes	55 (49.11)	41 (42.71)		
**Diabetes history**				
No	97 (86.61)	86 (89.58)	0.433	0.511
Yes	15 (13.39)	10 (10.42)		
**Hyperlipidemia history**				
No	84 (75.00)	79 (82.29)	1.621	0.203
Yes	28 (25.00)	17 (17.71)		
Hospitalization days (days)	14.32 ± 3.07	14.85 ± 3.20	1.217	0.225
NIHSS score	14.45 ± 3.36	16.26 ± 4.05	3.523	0.001
GCS score	13.23 ± 1.32	11.67 ± 1.24	−8.80	< 0.001
WBC (10^9^/L)	13.04 ± 3.38	13.50 ± 4.30	0.863	0.389
ANC (10^9^/L)	10.44 ± 2.05	10.77 ± 2.35	1.082	0.281
ALC (10^9^/L)	1.02 ± 0.17	1.08 ± 0.28	1.897	0.059
AMC (10^9^/L)	0.72 ± 0.24	0.66 ± 0.29	−1.78	0.076
Hemoglobin (g/L)	123.76 ± 10.57	125.73 ± 12.12	1.252	0.212
Platelet count (10^9^/L)	222.51 ± 58.58	221.11 ± 66.70	0.161	0.872
Fasting blood glucose (mmol/L)	5.78 ± 0.95	5.84 ± 1.08	0.426	0.670
Creatinine (μmol/L)	67.13 ± 7.45	61.99 ± 8.32	−4.700	< 0.001
Urea nitrogen (mmol/L)	5.55 ± 1.12	5.26 ± 1.05	−1.96	0.057
hs-CRP (mg/L)	10.07 ± 2.52	12.24 ± 3.68	5.018	< 0.001
D-Dimer (mg/L)	1.12 ± 0.18	1.17 ± 0.22	1.802	0.073
FDP (mg/L)	4.11 ± 0.60	4.26 ± 0.78	1.566	0.119
**Hemorrhage location**				
Basal ganglia	97 (86.61)	58 (60.42)	13.964	0.003
Cerebral lobe	6 (5.36)	13 (13.54)		
Thalamus	5 (4.46)	21 (21.88)		
Cerebellum	4 (3.57)	4 (4.17)		
Hematoma volume (mL)	20.01 ± 6.15	23.65 ± 6.01	4.300	< 0.001
**Hematoma shape**				
Regular	53 (47.32)	25 (26.04)	9.987	0.002
Irregular	59 (52.68)	71 (73.96)		

### Comparison of HMOX1 relative expression between the good and poor prognosis groups

**Figure 2:**
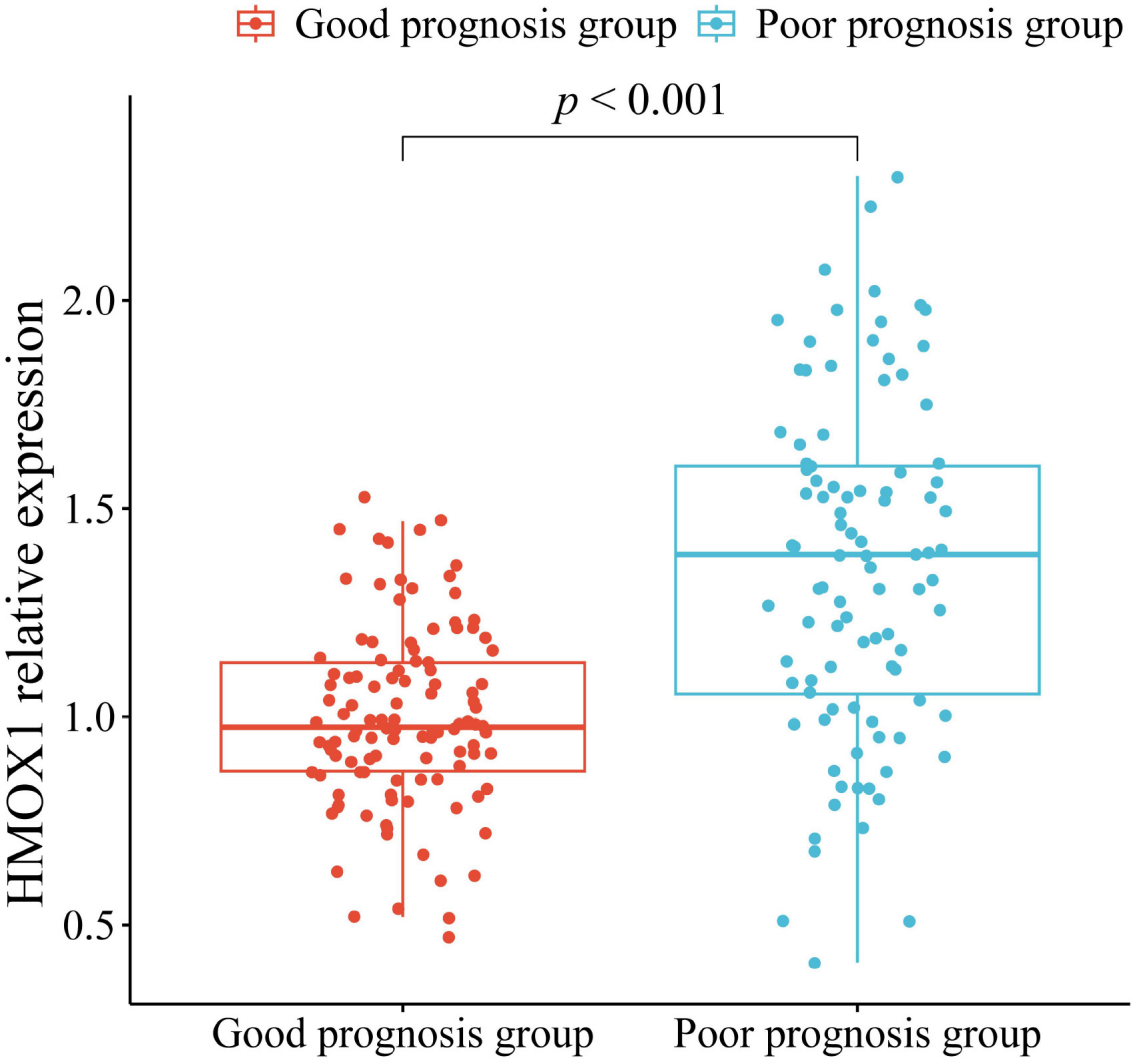
Box plot comparing HMOX1 mRNA expression between the good and poor prognosis groups.

The HMOX1 mRNA expression level in the poor prognosis group (1.36 ± 0.41) was significantly greater than that in the good prognosis group (1.00 ± 0.22) (*t* = 8.041, *P* < 0.001) (Figure 2).

### Hierarchical regression analysis of the relationships between clinical characteristics and mRS scores in HICH patients

In hierarchical 1, the mRS score was used as the dependent variable, and the NIHSS score was used as the independent variable for linear regression analysis. The results revealed that the NIHSS score had a significant positive effect on the mRS score (*t* = 3.212, *P* = 0.002). In hierarchical 2, GCS scores were added as an independent variable on the basis of hierarchical 1. The *R*^2^ value increased from 0.048 to 0.251, and the GCS score had a significant negative effect on the mRS score (*t* = −7.456, *P* < 0.001). Hierarchical lines 3, 4, 5, 6, and 8 included creatinine, hs-CRP, hemorrhage location, hematoma volume, and HMOX1 mRNA as independent variables, respectively. The results revealed that these indicators had a significant effect on the mRS score (*t* = −3.649, 4.773, 3.276, 2.928, and 6.044; all *P* < 0.05). Hierarchical 7 revealed that hematoma shape had no significant effect on the mRS score (*t* =1.533, *P* = 0.127) (Table 2).

**Table 2:** Hierarchical regression analysis of the relationships between clinical characteristics and mRS scores in HICH patients.

**Levels**	**Project**	**Unstandardized coefficient**		**β**	***t***	***P***	***R* ^2^**	***F***
		β	***SE***					
Level 1	Constant	1.022	0.428		2.390	0.018	0.048	10.316
	NIHSS score	0.087	0.027	0.218	3.212	0.002		
Level 2	Constant	7.268	0.921		7.900	< 0.001	0.251	34.318
	NIHSS score	0.057	0.024	0.143	2.339	0.020		
	GCS score	−0.463	0.062	−0.457	−7.456	< 0.001		
Level 3	Constant	9.319	1.056		8.828	< 0.001	0.297	28.693
	NIHSS score	0.061	0.024	0.152	2.547	0.012		
	GCS score	−0.424	0.061	−0.418	−6.923	< 0.001		
	Creatinine	−0.040	0.011	−0.218	−3.649	< 0.001		
Level 4	Constant	7.473	1.075		6.949	< 0.001	0.368	29.513
	NIHSS score	0.061	0.023	0.152	2.691	0.008		
	GCS score	−0.396	0.058	−0.392	−6.782	< 0.001		
	Creatinine	−0.038	0.010	−0.206	−3.640	< 0.001		
	hs-CRP	0.124	0.026	0.268	4.773	< 0.001		
Level 5	Constant	6.573	1.086		6.054	< 0.001	0.400	26.888
	NIHSS score	0.059	0.022	0.147	2.655	0.009		
	GCS score	−0.371	0.058	−0.366	−6.434	< 0.001		
	Creatinine	−0.036	0.010	−0.198	−3.561	< 0.001		
	hs-CRP	0.127	0.025	0.276	5.023	< 0.001		
	Hemorrhage location	0.321	0.098	0.181	3.276	0.001		
Level 6	Constant	5.522	1.125		4.909	< 0.001	0.424	24.676
	NIHSS score	0.054	0.022	0.136	2.498	0.013		
	GCS score	−0.357	0.057	−0.353	−6.296	< 0.001		
	Creatinine	−0.034	0.010	−0.183	−3.343	0.001		
	hs-CRP	0.125	0.025	0.271	5.032	< 0.001		
	Hemorrhage location	0.300	0.096	0.169	3.109	0.002		
	Hematoma volume	0.038	0.013	0.160	2.928	0.004		
Level 7	Constant	5.238	1.136		4.610	< 0.001	0.431	21.628
	NIHSS score	0.055	0.022	0.137	2.523	0.012		
	GCS score	−0.347	0.057	−0.343	−6.092	< 0.001		
	Creatinine	−0.033	0.010	−0.179	−3.280	0.001		
	hs-CRP	0.123	0.025	0.266	4.932	< 0.001		
	Hemorrhage location	0.293	0.096	0.166	3.053	0.003		
	Hematoma volume	0.037	0.013	0.155	2.859	0.005		
	Hematoma shape	0.260	0.170	0.083	1.533	0.127		
Level 8	Constant	2.892	1.117		2.590	0.010	0.519	26.854
	NIHSS score	0.051	0.020	0.129	2.574	0.011		
	GCS score	−0.259	0.054	−0.256	−4.751	< 0.001		
	Creatinine	−0.032	0.009	−0.173	−3.448	0.001		
	hs-CRP	0.105	0.023	0.228	4.563	< 0.001		
	Hemorrhage location	0.293	0.089	0.166	3.312	0.001		
	Hematoma volume	0.033	0.012	0.139	2.761	0.006		
	Hematoma shape	0.27	0.156	0.073	1.452	0.148		
	HMOX1 mRNA	1.311	0.217	0.316	6.044	< 0.001		

### Multivariate logistic regression analysis of factors affecting poor prognosis in HICH patients

Prior to conducting multivariate logistic regression, the variance inflation factor (VIF) was used to assess multicollinearity among all included predictors. Results showed that all VIF values were < 2, indicating no significant multicollinearity (Supplementary Table S1), which justified the inclusion of these factors in the regression model.

Indicators with statistically significant differences between the good and poor prognosis groups were selected as independent variables, while patient prognosis (coded as 0 = good prognosis, 1 = poor prognosis) served as the dependent variable. Multivariate logistic regression analysis revealed that increased NIHSS scores, elevated hs-CRP levels, cerebral lobe or cerebellar hemorrhage, larger hematoma volume, and higher HMOX1 mRNA expression were independent risk factors for poor prognosis (all P < 0.05). In contrast, higher GCS scores and elevated creatinine levels were identified as protective factors against poor prognosis (both *P* < 0.05) (Table 3).

**Table 3:** Multivariate logistic regression analysis of factors affecting poor prognosis in HICH patients.

**Indicator**	**β**	**SE**	**Wald χ^2^**	***P***	**OR (95% CI)**
NIHSS score	0.188	0.068	7.699	0.006	1.207 (1.057–1.378)
GCS score	−0.761	0.190	16.071	< 0.001	0.467 (0.322–0.678)
Creatinine	−0.103	0.034	9.266	0.002	0.902 (0.845–0.964)
hs-CRP	0.279	0.076	13.448	< 0.001	1.322 (1.139–1.535)
Hemorrhage location			9.630	0.022	
Basal ganglia					1.000
Cerebral lobe	2.156	1.088	3.927	0.048	8.637 (1.024–72.852)
Thalamus	1.334	0.730	3.334	0.068	3.795 (0.907–15.887)
Cerebellum	1.589	0.759	4.388	0.036	4.901 (1.108–21.681)
Hematoma volume	0.114	0.040	8.007	0.005	1.120 (1.036–1.212)
**Hematoma shape**					
Regular					1.000
Irregular	−0.742	0.493	2.270	0.132	0.476 (0.181–1.250)
HMOX1 mRNA	3.656	0.804	20.671	< 0.001	38.697 (8.003–187.120)

Notably, although the proportion of patients with cerebellar hemorrhage showed no statistically significant difference between the two prognosis groups in Table 1 (χ^2^ = 0.042, *P* = 0.837), multivariate analysis—after adjusting for confounding factors—demonstrated that cerebellar hemorrhage was associated with an odds ratio (OR) of 4.901 (*P* = 0.036). This finding suggests that the independent prognostic risk of cerebellar hemorrhage stems primarily from its high pathogenic severity (e.g., potential for brainstem compression and obstructive hydrocephalus) rather than its incidence frequency.

The logit equation for calculating the probability of poor prognosis (*P*) was as follows: logit (*P*) = 3.195 + 0.188 ^*^ NIHSS Score – 0.761 ^*^ GCS Score – 0.103^*^Creatinine + 0.279 ^*^ hs-CRP + 1.589 ^*^ Cerebellum (hemorrhage) + 1.334 ^*^ Thalamus (hemorrhage) + 2.156 ^*^ Cerebral Lobe (hemorrhage) + 0.114 ^*^ Hematoma Volume + 3.656 ^*^ HMOX1 mRNA.

1000 stratified Bootstrap resamples (stratified by prognosis) verified parameter stability. Key factors (e.g., HMOX1 mRNA, hemorrhage location) had small Bootstrap bias (< 12% of original coefficients), significant *P*-values (all *P* < 0.05), and concentrated 95% CIs. Original regression coefficients were close to the Bootstrap median, confirming reliable parameter estimation (Supplementary Table S2).

### Predictive value assessment of HMOX1 mRNA and imaging parameters

**Figure 3:**
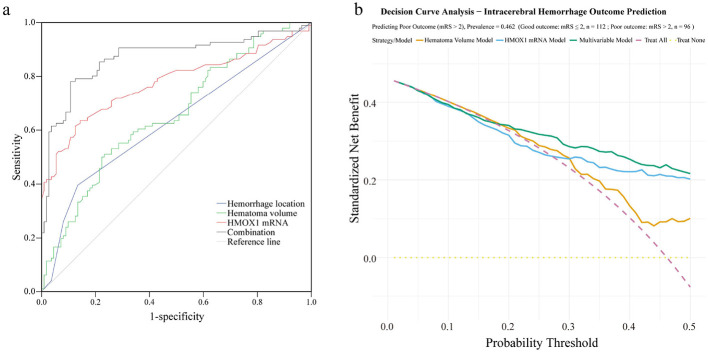
Prognostic value of HMOX1 mRNA and imaging parameters in HICH. **(a)** ROC curve analysis of predictive parameters; **(b)** comparison of decision curves between the nomogram model and other strategies.

When HMOX1 mRNA expression levels were combined with imaging parameters for prognosis prediction, Delong test was used to compare the AUC differences between the combined indicator and single indicators. The results showed that the AUC of the combination of HMOX1 mRNA and imaging parameters was 0.871 (95% CI: 0.818 – 0.924), which was significantly higher than that of hemorrhage location alone (AUC = 0.630, 95% CI: 0.552 – 0.707; AUC difference = 0.241, *P* < 0.001), hematoma volume alone (AUC = 0.657, 95% CI: 0.583 – 0.731; AUC difference = 0.214, *P* < 0.001), and HMOX1 mRNA alone (AUC = 0.775, 95% CI: 0.708 – 0.843; AUC difference = 0.096, *P* = 0.012) (Figure 3a, Table 4). These results confirm that the combined prediction of HMOX1 mRNA and imaging parameters has significantly better efficiency than single indicators, which highlights the advantages of combined prediction in improving prediction accuracy and sensitivity.

**Table 4:** Comparison of AUCs for HMOX1 mRNA, imaging parameters, and their combination (Delong test).

**Predictive indicator**	**AUC (95% CI)**	**Sensitivity (%)**	**Specificity (%)**	**AUC difference**	***P-*value**
HMOX1 mRNA + imaging parameters	0.871 (0.818–0.924)	79.12	90.02	–	–
Hemorrhage location alone	0.630 (0.552–0.707)	48.21	73.58	0.241	< 0.001
Hematoma volume alone	0.657 (0.583–0.731)	57.08	71.83	0.214	< 0.001
HMOX1 mRNA alone	0.775 (0.708–0.843)	64.28	85.94	0.096	0.012

Quantitative calibration verification via Hosmer–Lemeshow (H–L) test showed that the model had excellent calibration: H–L χ^2^ = 2.899, degrees of freedom = 8, *P* = 0.941 (*P* > 0.05), indicating no significant difference between the predicted probability of poor prognosis and the actual occurrence probability. To further confirm the superiority of the nomogram model, Delong test was performed to compare its AUC with that of the ‘HMOX1 mRNA + imaging parameters' combined indicator. The results showed that the AUC of the nomogram model (0.94, 95% CI: 0.90–0.97) was significantly higher than that of the combined indicator (AUC = 0.871, 95% CI: 0.818–0.924; AUC difference = 0.069, *P* = 0.003), indicating that the nomogram model has more excellent prognostic prediction efficiency (Table 5). DCA confirmed the clinical net benefit of the nomogram-based multivariable model. Within the clinically relevant threshold probability range (0.01–0.5), the multivariable model's net benefit was consistently higher than that of HMOX1 mRNA alone, hematoma volume alone, and extreme strategies. For example, at a threshold probability of 0.2 (key for intensive monitoring), the multivariable model's net benefit (0.340) was higher than HMOX1 mRNA model (0.315) and hematoma volume model (0.333); at 0.3 (threshold for surgical consultation), the multivariable model's net benefit (0.286) remained superior (Figure 3b, Supplementary Table S3).

**Table 5:** Comparison of AUCs between the nomogram model and the “HMOX1 mRNA + imaging parameters” combined indicator (Delong test).

**Predictive model**	**AUC (95% CI)**	**Sensitivity (%)**	**Specificity (%)**	**AUC difference**	***P-*value**
Nomogram model	0.94 (0.90–0.97)	84.26	90.4	–	–
HMOX1 mRNA + imaging parameters	0.871 (0.818–0.924)	79.12	90.02	0.069	0.003

### Evaluation of the prediction efficiency of the nomogram model

**Figure 4:**
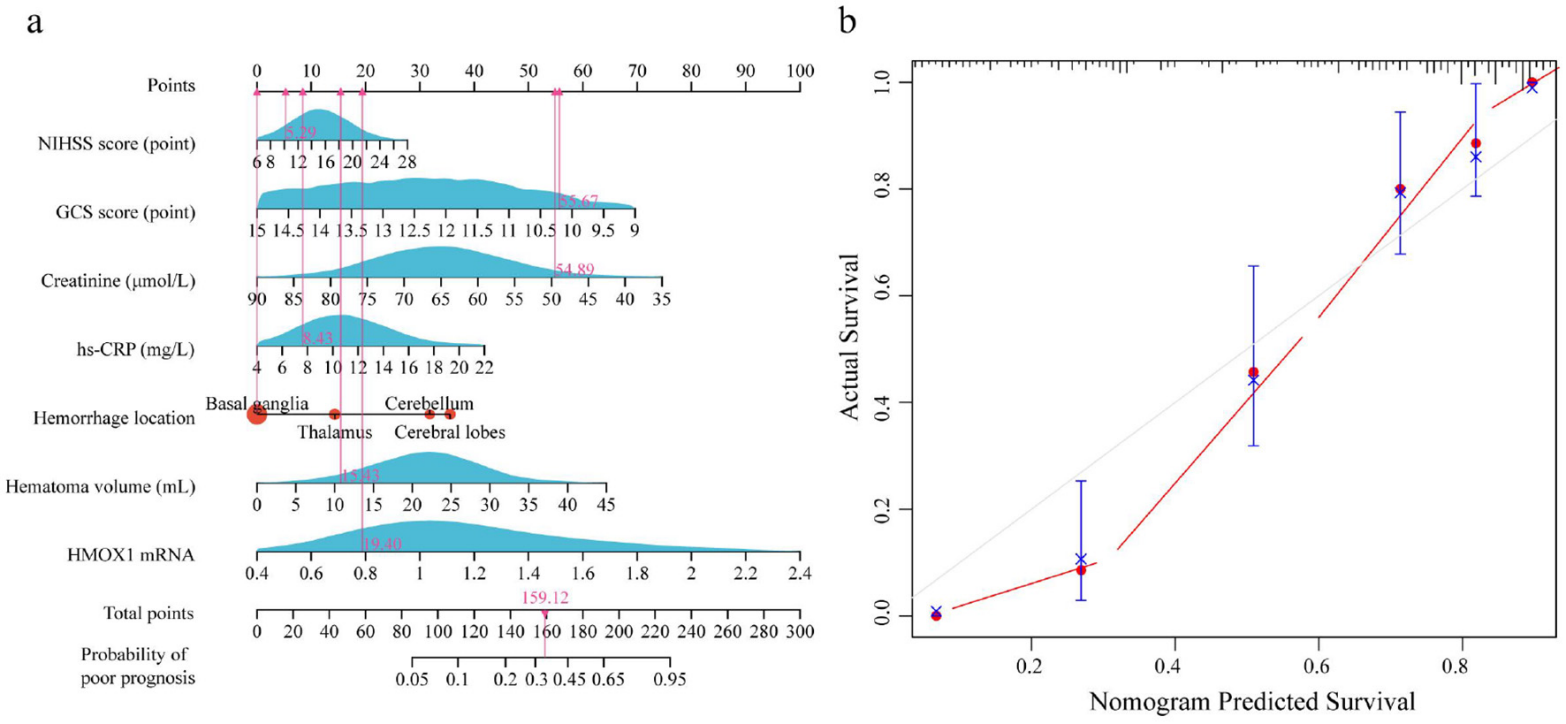
Evaluation of the nomogram model for predicting poor prognosis in HICH patients. **(a)** Nomogram model for prognostic prediction; **(b)** calibration curve for the nomogram model.

A nomogram model for predicting poor prognosis in HICH patients was constructed using the NIHSS score, GCS score, creatinine level, hs-CRP level, hemorrhage location, hematoma volume, and HMOX1 mRNA level as predictive factors (Figure 4a). The C-index of the nomogram model was 0.814 (95% CI: 0.778–0.850). Calibration curve analysis revealed that the calibration curve had good consistency with the ideal curve where the predicted probability was equal to the actual probability (Figure 4b).

## Discussion

This study explored the predictive value of HMOX1 mRNA combined with early imaging parameters for the prognosis of HICH patients. Through hierarchical regression analysis, multivariate logistic regression analysis, and ROC curve analysis, we systematically evaluated the role of key indicators, such as HMOX1 mRNA expression level, hemorrhage location, and hematoma volume, in predicting the prognosis of HICH patients and constructed a nomogram model for the comprehensive assessment of prognosis risk.

Our study revealed that HMOX1 mRNA expression levels were significantly higher in the poor prognosis group than in the good prognosis group, and hierarchical regression analysis revealed that HMOX1 mRNA had a significant positive effect on the mRS score. Multivariate logistic regression analysis revealed that HMOX1 mRNA expression was an independent risk factor for poor prognosis in HICH patients. This finding is consistent with previous studies on the role of ferroptosis in secondary brain injury after cerebral hemorrhage (11). As a key enzyme in heme metabolism, the dual role of HMOX1 may explain its complex relationship with prognosis. On the one hand, the biliverdin and carbon monoxide generated by HMOX1 catalysis have significant antioxidant and anti-inflammatory effects, reducing secondary damage to perihematomal tissue (12). On the other hand, free iron, a catalytic product, may exacerbate neurological damage by promoting ferroptosis (13). It is important to note that HMOX1 is a broad-spectrum stress-responsive gene, and its upregulation in peripheral blood can be induced by multiple pathways, including inflammation, oxidative stress, and ischemia, all of which are active in the pathological process of ICH. Therefore, the high HMOX1 mRNA expression observed in our study should be interpreted as a marker of intensified overall stress response rather than exclusively indicative of iron overload. While the association we observed is consistent with the ferroptosis mechanism demonstrated in animal experiments (7, 14), our clinical data cannot directly confirm a causal role for iron-dependent neurotoxicity. The precise mechanistic contribution of HMOX1 in human ICH requires further validation through studies correlating its expression with direct markers of ferroptosis and other pathological processes.

Hemorrhage location and hematoma volume are recognized as important factors affecting the prognosis of HICH patients (15–17). Our study found that patients with hemorrhage in the cerebral lobe and cerebellum and increased hematoma volume had a significantly greater risk of poor prognosis. This is consistent with previous studies (18, 19), indicating that the degree of compression and destruction of surrounding brain tissue by hematoma is one of the key factors determining patient prognosis. In particular, cerebral lobe and cerebellum hemorrhages, due to their proximity to key neural structures, can lead to severe neurological deficits after bleeding, thereby affecting patient prognosis (20–22). In our study, the nomogram assigned a higher score to cerebellar hemorrhage than to thalamic or basal ganglia hemorrhage, primarily because of its unique anatomical location and associated pathophysiological risks. The cerebellum is located in the posterior cranial fossa, with its ventral side directly adjacent to the brainstem (midbrain, pons, and medulla oblongata), which is the regulatory center for basic life functions such as respiration, circulation, and consciousness (23). Even a small hematoma volume in the cerebellum (e.g., < 5ml) can cause brainstem dysfunction through direct compression or the transmission of edema. For instance, compression of the pons' respiratory center can lead to abnormal respiratory rhythm, and damage to the medulla oblongata's cardiac center can result in circulatory failure (16). Moreover, the posterior cranial fossa has limited volume (about 1/5 of the cranial cavity), and a hematoma or secondary edema from cerebellar hemorrhage can easily cause a sudden increase in pressure in the posterior cranial fossa, leading to cerebellar tonsillar herniation. The herniated tissue can directly compress the medullary obex (a core area of the respiratory center) (24). In contrast, the basal ganglia and thalamus are located deep within the cerebral hemispheres, surrounded by ample compensatory space, and thus have a significantly lower risk of causing brain herniation with the same volume of bleeding. Therefore, the nomogram assigns a higher score weight to cerebellar hemorrhage. The superiority of the combined prediction and nomogram model was not only reflected in the numerical difference of AUC, but also verified by Delong test. Statistically significant differences confirmed that the 'HMOX1 mRNA + imaging parameters' combined indicator could overcome the limitation of single indicators in capturing pathological information, and the nomogram model, by further integrating clinical (NIHSS, GCS), laboratory (creatinine, hs-CRP) and molecular-imaging indicators, could more comprehensively assess the prognosis risk of HICH patients. This rigorous statistical verification avoids the bias of judging model advantages only by AUC values, and provides a more reliable basis for the clinical application of the nomogram model.

To comprehensively assess the risk of poor prognosis in HICH patients, this study constructed a nomogram model containing seven predictive factors: the NIHSS score, GCS score, creatinine level, hs-CRP level, hemorrhage location, hematoma volume, and HMOX1 mRNA. By comprehensively evaluating the patient's clinical characteristics, inflammatory status, renal function, and imaging parameters, the model achieved precise stratification of prognosis risk in HICH patients. The C-index of the nomogram model was 0.814, and the AUC was 0.94, indicating good discrimination and calibration. The clinical value of a predictive model is determined not only by its accuracy but also by its practical utility and comparative advantage over existing tools. Numerous prognostic models for ICH have been developed, primarily based on clinical scores (e.g., GCS, NIHSS) and CT imaging features such as hematoma volume and location (9, 10, 16, 18). The distinct advantage of our nomogram lies in its integration of a molecular biomarker (HMOX1 mRNA) with these established clinical and radiological parameters. This multi-modal approach aims to capture a broader spectrum of the underlying pathophysiology, including the crucial role of oxidative stress and inflammation implicated in secondary brain injury. This likely contributes to the model's high predictive accuracy (AUC: 0.94), which, as confirmed by the Delong test, was significantly superior to the combination of imaging and HMOX1 alone and to models relying on single parameter classes.

In summary, HMOX1 mRNA combined with early imaging parameters has important value in predicting the prognosis of HICH patients. By constructing a combined prediction model, we can more comprehensively assess patient prognosis risk, providing a scientific basis for developing individualized treatment plans. Doctors can adjust treatment plans on the basis of the patient's prognosis risk score to improve treatment outcomes and patient prognosis. Although this study has achieved certain results, it has several limitations that need to be addressed. The selection of study subjects is somewhat restrictive. We only included HICH patients undergoing conservative treatment and excluded those who received surgical treatment or abandoned treatment/died rapidly during hospitalization. It is worth noting that the included patients had a mean age of 50 ± 9.5 years, which is lower than the typical average age (usually ≥60 years) of ICH cohorts. This discrepancy is determined by the demographic characteristics of the region served by the research center—he study service area has a high proportion of young people, and elderly HICH patients tend to be referred elsewhere, resulting in insufficient representation of the elderly HICH population in the sample. Given that elderly ICH patients often have multiple underlying diseases such as diabetes and coronary heart disease, and have poorer brain reserve function, their prognostic influencing factors and responses to HMOX1 mRNA and imaging parameters may differ from those of middle-aged and young patients. Therefore, the applicability of the predictive model constructed in this study to elderly HICH patients undergoing conservative treatment needs to be carefully assessed. Fourth, our study utilized baseline neurological scores (NIHSS and GCS) and did not analyze their dynamic changes over time, which might contain additional prognostic information. Furthermore, the use of the total NIHSS score, while clinically validated, precludes insight into the contribution of specific subitems (e.g., consciousness). Future prospective studies incorporating serial assessments and subitem analysis could further refine prognostic models. Additionally, as a single-center study, the results need to be verified in multicenter studies to enhance the generalizability of the findings. We also did not dynamically monitor HMOX1 mRNA changes over time or include additional ferroptosis-related markers, which limits our understanding of the temporal dynamics of HMOX1 mRNA expression and the underlying mechanisms. Despite these limitations, the study employed rigorous internal validation techniques, including the Hosmer–Lemeshow test, DCA, and stratified Bootstrap resampling. In future research, we plan to conduct multicenter prospective studies to validate our findings in a broader patient population. We will also attempt to include more biomarkers and imaging parameters in the model to improve the comprehensiveness and accuracy of predictions. Moreover, we will focus on promoting and applying the prediction model in clinical practice to benefit more patients.

## Data Availability

The raw data supporting the conclusions of this article will be made available by the authors, without undue reservation.
